# Advancing health equity in cancer care through collaborative research agenda development: a provincial workshop guided by patient-oriented research in Alberta, Canada

**DOI:** 10.3389/fonc.2026.1937938

**Published:** 2026-10-15

**Authors:** Anna Pujadas Botey, Harkeert Judge, Paula J. Robson, Anna Santos Salas

**Affiliations:** 1 Research, Partnerships and Innovation, Cancer Care Alberta, Edmonton, AB, Canada; 2 School of Public Health, University of Alberta, Edmonton, AB, Canada; 3 Faculty of Nursing, University of Alberta, Edmonton, AB, Canada

**Keywords:** cancer care, co-design, community-engaged research, health equity, patient engagement, patient-oriented research (POR), research agenda development

## Abstract

Health inequities in cancer care persist, indicating that advancing equity requires not only understanding inequities but also transforming how cancer research is developed and conducted. Although equity-oriented and community-engaged approaches have advanced important principles for research, there is limited understanding of the conditions and processes required to translate these principles into practice within cancer care systems, including how research agendas can be collaboratively developed to reflect community and health system needs. This Perspective describes the co-design and implementation of a provincial Health Equity Workshop in Alberta, Canada, as a practice-based example of how a cancer care system operationalized Patient-Oriented Research principles to support equity-oriented research agenda development. Guided by Patient-Oriented Research and informed by the Health Equity Implementation Framework, the initiative involved a twelve-member working group comprising people with lived experience of cancer, representatives of community organizations, health system leaders, and researchers who co-designed and facilitated a two-day workshop involving 43 participants. Reflections from the initiative highlighted relational, organizational, and practical conditions that shaped equity-oriented engagement, including trust-building, shared leadership, compensation and resource supports, institutional commitment, and attention to system-level barriers affecting participation. The workshop demonstrated how co-design can embed equity from the earliest stages of research agenda development by bringing together diverse forms of expertise to shape what research is needed and how it should be conducted. This Perspective offers practical insights for researchers, health systems, and community partners seeking to advance equitable cancer research and care.

## 1 Introduction

Health equity, defined as the absence of avoidable and unfair differences in health and its determinants across population groups (1, 2), is a foundational goal in cancer care across Canada. Yet persistent disparities in access, experience, and outcomes indicate equity has not been realized (3, 4). Canadian studies have documented these inequities across multiple populations, including immigrants, racialized populations, and other underserved groups, and have highlighted the social and structural determinants that contribute to them (5–9). However, generating evidence about disparities and their underlying determinants alone is insufficient to advance equity. Achieving more equitable cancer care also requires intentional decisions about what research is undertaken, whose perspectives shape the research agenda, and how research is designed, conducted, and translated into practice (4).

Internationally, there is growing recognition that advancing health equity requires research processes that are collaborative, community-engaged, and responsive to lived experience (10–14). Across the United States, Canada, Australia, New Zealand, and elsewhere, complementary approaches such as patient-oriented research, community-based participatory research, integrated knowledge translation, and, more recently, equity-focused implementation science, have emphasized embedding equity throughout the research process (10–13). Collectively, these approaches emphasize power-sharing, meaningful involvement of patients and communities throughout the research process, transparent governance, equitable knowledge translation, and sustained partnerships as essential components of equity-oriented research (10, 12–14). Recent work further argues that equity should be embedded across all phases of the research process (from identifying research questions and designing studies through implementation, dissemination, and evaluation), and cautions against engagement that is symbolic rather than transformative (10, 11, 15–17).

Despite these advances, less attention has been paid to how these principles are operationalized in practice within cancer care systems. Although conceptual guidance and practice-based examples of equity-oriented research are increasing, relatively little is known about how key stakeholders across communities, health systems, and research environments work together to shape the direction of equity-oriented cancer research. As a result, there is limited understanding of the conditions and processes required to translate equity principles into cancer research practice. One important way to shape the direction of equity-oriented cancer research is through the collaborative development of a research agenda. This requires the concurrent engagement of patients and communities, health system leaders, and researchers, each contributing distinct forms of expertise needed to ensure that future research is rigorous, relevant, and responsive to community and health system needs (18–22).

Patient-Oriented Research (POR) provided the primary framework for this initiative because of its explicit emphasis on partnering with patients throughout the research process (18). By positioning patients as partners and co-producers of knowledge, POR emphasizes that lived experience should meaningfully inform what questions matter, how research is conducted, and how findings are interpreted and applied. Within POR, patients include individuals with lived experience of a health condition as well as their caregivers and close supporters (18). Applied through an equity lens, POR also provides a foundation for meaningful partnerships with community members and organizations, service providers, decision-makers, and researchers whose perspectives are essential for understanding and addressing health inequities. Consistent with broader principles of community-engaged and equity-oriented research (10–13), this broader application of POR supports the collaborative development of research agendas that are responsive to lived experience and grounded in the realities of health system delivery.

In Alberta, Canada, Cancer Care Alberta (CCA), the provincial cancer program responsible for delivering cancer services within a population of approximately five million residents, recognized that advancing health equity required innovative ways of thinking and acting that extended beyond traditional quality improvement approaches. Consistent with its organizational commitment to “equity by design,” CCA partnered with people with lived experience of cancer, representatives of community organizations, and researchers to co-develop and co-lead a provincial Health Equity Workshop. Guided by an equity-oriented application of POR, the workshop brought together people with lived experience of cancer from racialized, rural, and gender-diverse communities, alongside representatives of community organizations, health system leaders, service providers, and researchers. The workshop aimed to create a safe space in which participants could collaboratively shape discussions that informed the development of an equity-oriented cancer research agenda.

This Perspective describes the co-design and implementation of a provincial Health Equity Workshop as a practice-based reflection on how a cancer care system operationalized POR principles to support the collaborative development of an equity-oriented cancer research agenda. Rather than proposing a new framework, this Perspective makes visible relational, organizational, and practical conditions involved in translating equity-oriented and POR principles into practice. It examines how these conditions shaped equity-oriented engagement and discusses their implications for researchers, health systems, and community partners.

## 2 Operationalizing equity through collaborative research agenda development

### 2.1 Conceptual foundations

Consistent with the broader principles of community-engaged and equity-oriented research (10–13), this initiative was guided primarily by the principles of POR (18) and informed by the Health Equity Implementation Framework (HEIF) (23, 24). POR was selected because of its emphasis on partnering with people with lived experience throughout the research process and its application as a foundation for meaningful partnerships among community members, health system partners, and researchers (18), while HEIF provided a complementary framework for examining the social, structural, and health system factors that influence equity in cancer care.

POR informed how the workshop was designed and conducted by guiding partnership, shared decision-making, facilitation, and reflection throughout the process. Rather than treating engagement as a discrete activity, POR shaped who was involved, how questions were framed, how dialogue was facilitated, how decisions were made, and how diverse forms of knowledge were recognized and valued. HEIF informed what participants explored by guiding discussion of these social, structural, and health system factors throughout the workshop. Together, these frameworks positioned equity not only as a topic of discussion but also as a way of working that shaped both the collaborative agenda development process and the insights that emerged.

### 2.2 Partnership model

The workshop was co-designed through a partnership model that brought together a twelve-member working group comprising patients, survivors and caregivers with lived experience from racialized, rural, and gender-diverse communities; members of community organizations; CCA health system leaders; and researchers. The term survivor was used to describe any individual who has received a cancer diagnosis, spanning from the time of initial diagnosis through the remainder of their life, regardless of their current treatment status. This includes individuals actively undergoing therapy, those on long-term maintenance, and those who have completed primary treatments (25, 26). The group was intentionally constituted to bring together diverse roles, lived experiences, and forms of expertise relevant to the co-design process, including patient partners with lived experience connected to the communities of interest. Demographic information was not systematically collected from working group members, which limits out ability to characterize the demographic composition of the working group. Together, the group worked to advance health equity in cancer care. Meetings were coordinated by three working group co-chairs, including representatives from the academic and health system partners, while facilitation and decision-making responsibilities were shared across the working group rather than concentrated in the co-chairs. Working group members collectively shaped meeting agendas, contributed to discussions, and participated in decisions regarding workshop goals, content, structure, recruitment, facilitation and follow-up. Decisions were made through discussion and consensus rather than by a single designated lead.

The endorsement and active participation of CCA leadership created a direct link between community-identified insights and system-level decision-making. This demonstrated how institutional leaders can support shared decision-making with communities while ensuring that equity-informed discussions are connected to organizational planning and future research.

### 2.3 Workshop design

The Health Equity Workshop was held in September 2025 in Enoch, Alberta, a traditional territory of First Nations peoples neighbouring the city of Edmonton. The workshop consisted of a two-day, in-person event designed to support relational, trust-based engagement, particularly with communities experiencing inequities. It brought together 43 participants, including people with lived experience of cancer care (patients, survivors, and caregivers), representatives of community organizations, health system leaders, service providers, and academic partners from CCA and the University of Alberta. Building on existing community connections, we invited people from racialized communities, rural areas of Alberta, and gender-diverse communities, as well as community organization leaders and advocates connected to these communities. These participants brought diverse lived experiences of cancer care as patients, survivors, and caregivers, as well as community perspectives. Individual and community organization participants were invited based on their connection to the communities of interest experiencing inequities in cancer care, lived experience of cancer care, and willingness and ability to contribute their experiences. Demographic information was not systematically collected from workshop participants, which limits our ability to provide a detailed demographic characterization of the participants or quantify representation across specific racialized, rural, gender-diverse, or other sociodemographic groups.

All participants engaged in discussions as partners, contributing different forms of expertise (18, 22). Sessions were organized around exploring research needs across three thematic areas, selected by the working group and informed by an established framework reflecting components of cancer care equity (27) (1): addressing social determinants of health (2); advancing equitable access to high-quality care; and (3) ensuring equitable access to research. The workshop began with an Indigenous Elder-led opening prayer and smudging ceremony, setting a tone of respect, inclusion, and cultural awareness.

Each session included a panel discussion and small-group, roundtable discussions, with time for lived-experience stories, reflection and art. Shared meals and frequent breaks were provided to create opportunities for informal conversations outside formal sessions. To support equitable participation, patient and community participants received compensation and full coverage for travel and accommodation.

### 2.4 Immediate outcomes

The workshop surfaced values, tensions, and shared aspirations that can inform future directions for equity-oriented cancer research. Discussions highlighted tensions between community expectations for flexible and sustained engagement and institutional structures shaped by fixed funding cycles, organizational boundaries, and existing processes; between community-defined needs and health system and research perspectives on how areas of focus for future research should be identified and framed; and among the diverse experiences and perspectives of racialized, rural, and gender-diverse communities. Discussions also highlighted how governance structures that do not adequately include community perspectives can constrain whose knowledge and perspectives shape research decisions. These tensions did not necessarily reflect disagreement, but rather differences in how equity, participation, and research needs were understood across contexts. Bringing these perspectives together made visible some of the practical and organizational challenges that health systems must navigate when seeking to operationalize equity. This workshop therefore served as a first step in building ongoing relationships and demonstrating how health systems can collaborate with communities experiencing inequities to shape research directions that reflect their lived experiences, needs, and perspectives.

The workshop also identified areas of focus that can inform future research and action across the three thematic domains. These included reimagining geographic boundaries of cancer care, increasing financial supports, and advancing culturally relevant care; building trusting relationships in cancer care, navigating access to care across the cancer journey, strengthening cancer care teams, and understanding delays in access to cancer care; and addressing gaps in cancer research awareness, understanding, and trust, and cultivating meaningful inclusion in research. These areas of focus will continue to be refined through ongoing engagement with patients, community members, and CCA leaders and researchers. A separate manuscript, developed with the broader working group, describes the process through which these areas of focus were identified and refined and is currently in development.

## 3 Practice-based reflections on equity in practice

This section presents practice-based reflections on how equitable engagement can be operationalized in cancer research, synthesized by the authors from the workshop and co-design process. Four of these reflections are presented as practice-based lessons (Sections 3.1-3.4). These reflections were informed by working group discussions, workshop documentation, and post-workshop reflection, and were subsequently discussed with members of the working group during manuscript development. Together, they capture recurring observations about the relational, organizational, and practical conditions that shaped equity-oriented engagement and are presented as practice-based reflections rather than as formally derived qualitative findings. A final subsection (Section 3.5) presents an additional practice-based reflection on culturally responsive and accessible approaches to engagement that emerged from the workshop and co-design process.

### 3.1 Lesson 1: equity requires time and trust

Building equitable partnerships cannot be rushed or reduced to mere consultation or engagement. Trust is developed through time, consistency, and genuine listening. The workshop deliberately included shared meals, frequent breaks, opportunities for reflection, laughter, and informal conversation alongside formal sessions. These spaces created opportunities for participants to connect beyond their formal roles and, as documented during the workshop and in subsequent post-workshop reflection, contributed to an environment in which participants could feel safe sharing sensitive experiences and engage in open, honest dialogue. Across discussions, trust was repeatedly identified as foundational to equity, shaping both who participated and the depth of dialogue that was possible. Building equitable partnerships also requires recognition that trust cannot be assumed. Historical experiences of exclusion, exploitation, and harm have shaped how some communities engage with research and health institutions, underscoring the importance of transparency, reciprocity, and demonstrated commitment to community partnership (28).

### 3.2 Lesson 2: co-design is equity work

Equity depends on who helps shape the work. By involving patient and community members as co-leads in planning, decision-making, and facilitation, co-design served as one of the mechanisms through which POR principles were put into practice alongside broader equity-oriented engagement approaches. The process itself modeled inclusivity, shared ownership, and reciprocal learning, moving engagement beyond tokenism toward genuine partnership. By embedding equity throughout the workshop process, this approach laid the foundation for the collaborative development of an equity-oriented cancer research agenda, which can ultimately contribute to more equitable care. This was reflected in practical decisions about the workshop structure, facilitation, recruitment, and participant supports, which were discussed and agreed upon through the working group rather than determined solely by researchers or health system leaders.

Importantly, equitable co-design required the intentional involvement of health system partners who were willing to participate with humility, openness, and a readiness to listen rather than defend existing practices. Attention to who represents the system, and how they show up, helped mitigate power imbalances and supported more honest and productive dialogue.

### 3.3 Lesson 3: power-sharing and compensation are structural, not symbolic

Equity requires tangible commitments. During the workshop, providing compensation, covering travel and accommodation, and involving patient and community members in planning, facilitation, and decision-making were intended to recognize the value of their expertise and participation and to reduce practical barriers to participation that might otherwise have limited whose perspective could be included. Participants emphasized that community organizations already hold contextual knowledge essential for equity research, reinforcing the importance of recognizing, valuing, and resourcing the collective expertise of communities outside academic and institutional settings.

### 3.4 Lesson 4: system structures can limit inclusion in research

Participants’ experiences and discussions highlighted how existing system structures can constrain meaningful engagement in research, particularly for rural, racialized, and gender-diverse communities. Limited demographic data, institutional silos, and inflexible funding and organizational structures can restrict the ability of health systems to respond to community-identified needs and sustain relationships beyond existing processes and timelines. These experiences highlighted that creating opportunities for participation alone is insufficient when the structures within which research is planned and funded limit how community perspectives can influence decisions. Without system-level flexibility, even well-intentioned initiatives risk reproducing inequities rather than transforming them.

### 3.5 Additional reflection: people-centered approaches to engagement

Observations from the workshop and co-design process highlighted the importance of culturally responsive and accessible approaches to engagement. Intentional design and facilitation choices, including an Indigenous Elder-led opening prayer and smudging ceremony, co-facilitation, plain language, art, reflection, and opportunities for informal connection, reinforced that operationalizing equity is both relational and practical. The way the workshop was designed and facilitated also shaped the process of collaboratively developing an equity-oriented research agenda, influencing whose perspectives informed discussions, how ideas were explored and refined, and which approaches were considered feasible and meaningful for diverse communities. These choices illustrate how equitable engagement practices can guide research agenda development and help ensure that community perspectives inform both the direction of future research and how it is designed and conducted.

An important reflection was the extent to which participants across diverse communities articulated shared challenges and concerns. While experiences differed, common themes (such as travel burden, fear of discrimination, and reliance on family support) cut across racialized, gender-diverse, and rural contexts. Many participants occupied multiple social positions, highlighting the intersectional nature of inequity and underscoring the importance of research approaches that are responsive to lived realities (29). Participants also expressed the importance of learning from members of communities other than their own.

## 4 Discussion

Our reflections are consistent with a growing international movement toward community-engaged and equity-oriented cancer research. Across POR, community-based participatory research, integrated knowledge translation, and equity-focused implementation science, researchers have emphasized that advancing equity requires power-sharing, meaningful engagement of patients and communities, transparent governance, and sustained partnerships throughout the research process (10–12, 14–17, 30, 31). Although this literature provides strong conceptual guidance, fewer publications describe how cancer care systems operationalize these principles in practice. Our experience highlights three interrelated dimensions that shaped the operationalization of equity-oriented research in this setting: relational conditions that supported trust and shared learning, organizational conditions that enabled meaningful participation, and practical conditions that shaped how collaboration occurred. This Perspective contributes a practice-based example of how a cancer care system operationalized POR principles through a co-designed process to collaboratively develop an equity-oriented research agenda involving people with lived experience, community organizations, health system leaders, service providers, and researchers. Rather than proposing a new framework, we make visible the relational, organizational, and practical work required to translate equity principles into research practice.

Taken together, our four lessons, complemented by the additional reflection on people-centered approaches, demonstrate that operationalizing equity within cancer research requires more than meaningful engagement alone. It depends on sustained partnerships in which communities, researchers, and health system leaders work together to develop research agendas that are responsive to community needs and priorities. Developing an equity-oriented research agenda depends on creating the relational conditions that support trust and shared learning, establishing organizational conditions that redistribute power and resources, and addressing the practical conditions that enable meaningful participation. In this way, equity becomes not only an outcome of research, but also a defining characteristic of how research is designed and conducted.

### 4.1 Operationalizing equity requires both relational and organizational change

Operationalizing equity depends on creating both relational and organizational conditions that enable meaningful partnership. Trust-building, cultural humility, reciprocal learning, and shared decision-making created the relational conditions that enabled participants to contribute openly and shape discussions. At the same time, organizational supports such as participant compensation, institutional leadership, shared governance, and dedicated resources signaled that community expertise was valued and created opportunities for meaningful participation. Importantly, these relational and organizational conditions were not secondary to the workshop. They shaped who participated, how research directions were discussed, and which research questions emerged. Together, these relational and organizational conditions shifted engagement beyond consultation toward partnership. This distinction is important because equitable research depends not only on who is invited to participate, but also on whether the research environment enables communities to influence research agendas and decision-making.

### 4.2 Implications for researchers, health systems, and community partners

Researchers should design and evaluate equitable engagement not only by documenting who participates but also by examining how decisions, responsibilities, and benefits are shared throughout the research process (10, 11). Health systems have an equally important role in creating the structural conditions that make equitable partnerships possible through flexible funding, shared governance, institutional leadership, and long-term investment in community relationships (10, 12, 32). Patients and community organizations should be recognized as partners whose expertise extends beyond consultation to shaping research agendas, informing study design, interpreting findings, and guiding implementation. Together, these actions can strengthen the relevance, legitimacy, and responsiveness of equity-oriented cancer research.

### 4.3 Future directions

This workshop represents one example of how a cancer care system can operationalize equity-oriented research agenda development. Future work should examine how these partnership models influence subsequent research agendas, funding decisions, implementation strategies, and ultimately cancer care outcomes. Longitudinal evaluation will be particularly important for understanding whether investments in trust-building, shared governance, and community leadership translate into sustained changes in research agendas, funding priorities, and health system practice. Evaluating the transferability of similar partnership models across different cancer care systems, healthcare, and international contexts will also help determine how these approaches can be adapted to diverse settings.

## 5 Conclusion

Advancing health equity in cancer care depends not only on understanding inequities, but also on transforming how equity-oriented cancer research agendas are developed and who is involved in shaping them. Although conceptual guidance for equity-oriented research continues to expand, fewer examples describe how these principles can be operationalized within cancer care systems. This Perspective provides one such example by illustrating how a cancer care system operationalized POR principles through a co-designed process that laid the foundation for the collaborative development of an equity-oriented cancer research agenda involving people with lived experience of cancer, community organizations, health system leaders, service providers, and researchers. By making explicit the relational, organizational, and practical conditions that support collaborative research agenda development, this Perspective contributes to the growing literature on embedding equity within research practice while offering a practical example for cancer care systems seeking to translate commitments to equity into research practice.
